# Splenic flexure volvulus: a rare case report

**DOI:** 10.1093/jscr/rjac354

**Published:** 2022-07-31

**Authors:** Landry Umbu, Swathi Muttana, Karimah Best, Nadia Kamagate, Peter DeVito

**Affiliations:** Department of Surgery, Trumbull Regional Medical Center, Warren, OH, USA; Department of Surgery, American University of Antigua, College of Medicine, New York, NY, USA; Department of Surgery, American University of Antigua, College of Medicine, New York, NY, USA; Department of Surgery, Trumbull Regional Medical Center, Warren, OH, USA; Department of Surgery, Trumbull Regional Medical Center, Warren, OH, USA

## Abstract

Colonic volvulus, where the colon twists around its mesentery, commonly occurs in the sigmoid and cecum. However, colonic volvulus of the splenic flexure is quite rare. Reported cases are limited but suggest that prolonged constipation in patients with either congenital anomalies, history of prior abdominal surgery, and or psychiatric history are described as common risk factors for large bowel volvulus. Here, we discuss a case of a 56-year-old man with a history of chronic constipation and no previous abdominal surgeries who presented to the emergency department with abdominal pain and distention. Further workup including a computed tomography imaging and decompressive via limited colonoscopy confirmed diagnosis of colonic volvulus of the splenic flexure. Surgical management of colonic volvulus is patient specific but invariably involves partial colectomy, as was performed in this case.

## INTRODUCTION

Intestinal volvulus entails a portion of the gastrointestinal tract rotates along its mesentery. The affected portion of the bowel can therefore be at increased risk for arterial and/or venous occlusion, and bowel obstruction. A volvulus can occur in any portion of the colon, where it is not attached to the retroperitoneum [1]. This is most commonly seen in the cecum, or sigmoid colon. A splenic flexure volvulus (SFV) is usually densely attached to its adjacent organs, making it the least common site to develop a colonic volvulus [2]. Given its rarity, this makes the condition difficult to diagnose, and subsequently result in delays in treatment.

SFV can be categorized into both primary and secondary etiologies. Both comprise of hypermobility of the splenic flexure with torsion around its mesentery due to mobilization of the descending colon from retroperitoneal attachments, as opposed to the absence or laxity of phrenicocolic, gastrocolic and/or splenocolic ligaments [3].

## CASE PRESENTATION

A 56-year-old male with history for chronic constipation, and no previous abdominal surgeries presented to the emergency department complaining of intermittent abdominal pain and distention. His vital signs were within normal limits. On examination, the abdomen was noted to be distended, tympanic, non-tender without guarding or rebound tenderness.

Laboratory evaluation showed no significant leukocytosis and hemoglobin was also noted to be within normal limits.

**Figure 1:**
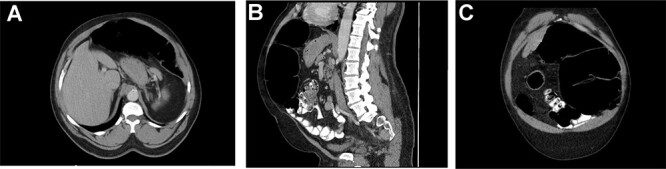
(**A**) showing distended transverse colon. (**B**) Sagittal displaying distended splenic flexure, concern for SFV. (**C**) Coronal view showing dilated splenic flexure, concern for SFV.

A computed tomography (CT) additionally revealed moderate distention of the transverse segment of colon (Fig. 1A), abnormal appearance of the left upper quadrant mesentery with whorled appearance of the splenic flexure (Fig. 1B), suggestive of partial obstruction and dilation of the colon at the level of the splenic flexure (Fig. 1C). The patient was admitted to the surgical floor for further management.

The patient underwent a decompressive colonoscopy, a segment of volvulus was decompressed at the area of the splenic flexure and transverse colon. After endoscopic decompression, the colon was then prepped for elective surgical resection. The patient underwent an open left hemicolectomy, end to end colo-colonic anastomosis and intraoperative sigmoidoscopy. The twisted splenic flexure was noted to be redundant, medially displaced, with proximal dilation of the transverse colon and cecum noticed.

Several thin adhesive bands were found, attaching the antimesenteric portion of the splenic flexure medially onto the colon mesentery. These adhesions were carefully taken down, restoring the splenic flexure to its normal position and the remainder of the bowel was thoroughly inspected prior to resection of the descending colon, no obvious masses or abnormalities were appreciated. Diet was slowly advanced with the return of bowel function. He was subsequently discharged to a skilled nursing facility for further recovery.

## DISCUSSION

Volvulus of the colon is the second most common cause of large bowel obstruction. The affected portion of bowel may become entirely or partially obstructed, and is at increased risk of ischemia [4]. In most instances, a volvulus occurs in segments of the colon that is fixed or attached to the retroperitoneum [1] and is most prevalent in either the sigmoid colon or cecum. At the level of the splenic flexure, the colon normally has fixed ligamentous attachments to the stomach, spleen and diaphragm. These fixed attachments make the splenic flexure a rare location for the development of volvulus, accounting for 2% of all colonic volvulus [2].

SFV can be classified as primary or secondary etiologies. Primary SVF is associated with congenital anomalies, including absence or laxity of splenic ligaments, congenital bands and a wandering spleen. Secondary etiologies of SFV comprise two-thirds of SFV cases, and are caused due to underlying disruption of ligaments, mobilization of the splenic flexure or adhesions from prior surgeries [1].

There are many risk factors associated with the development of SFV and they coincide with the primary and secondary etiologies. For individuals affected by a congenital anomaly, the main risk factor is chronic constipation, and others include Hirschprung’s disease, and cerebral palsy or myotonic dystrophy. In terms of the development of secondary SFV, the main predisposing cause is related to previous abdominal surgery involving the splenic flexure.

Patients present with sudden onset of large bowel obstruction, abdominal pain, distention, constipation, nausea and vomiting [2]. Physical examination may reveal a palpable abdominal mass and no stool in the rectal vault [2].

Three important components that can assist in diagnosis include: patient’s history, examination findings and radiological findings, with CT having the highest sensitivity and specificity [2, 5].

Plain abdominal X-ray typically reveals several characteristic features of SFV, including dilated large bowel loop with sudden termination at the splenic flexure [1, 2, 5]. If a barium enema is performed, a bird’s beak appearance can be seen at the splenic flexure. On a CT scan, twisting of the mesentery is demonstrated with a swirl and coffee bean sign [1, 2, 5].

The management of a SFV is dependent on clinical factors, mainly revolving around the presence or absence of peritonitis and or hemodynamic instability. If peritoneal signs are present, emergency surgery is required [3]. In the absence of peritonitis, a conservative approach is warranted, which consists of endoscopic detorsion following elective resection of the volvulus [2]. Resection of the splenic flexure is advised, given the high risk of recurrence. Surgical resection with primary anastomosis with or without diverting ileostomy, or resection with end colostomy is operative options that can be decided based on the patient’s clinical factors.

For high risk or elderly patients, there is a less invasive option known as colopexy.

## CONCLUSION

Splenic flexure volvulus is a very rare type of colonic volvulus usually seen in patients with chronic constipation or history of previous surgical mobilization. Identifying this disease process requires a high index of suspicion, as missed diagnosis can have detrimental consequences including bowel ischemia and perforation. Approach to managing this rare cause of colonic obstruction varies, depending on clinical presentation. Usual surgical intervention results in resection to decrease the risk of recurrence.
